# Screening for Intellectual Disability Using High-Resolution CMA Technology in a Retrospective Cohort from Central Brazil

**DOI:** 10.1371/journal.pone.0103117

**Published:** 2014-07-25

**Authors:** Rodrigo Roncato Pereira, Irene Plaza Pinto, Lysa Bernardes Minasi, Aldaires Vieira de Melo, Damiana Mirian da Cruz e Cunha, Alex Silva Cruz, Cristiano Luiz Ribeiro, Cláudio Carlos da Silva, Daniela de Melo e Silva, Aparecido Divino da Cruz

**Affiliations:** 1 Núcleo de Pesquisas Replicon, Departamento de Biologia, Pontifícia Universidade Católica de Goiás, Goiânia, Goiás, Brazil; 2 Programa de Pós-Graduação em Biologia Celular e Molecular, Universidade Federal de Goiás, Goiânia, Goiás, Brazil; 3 Programa de Pós-Graduação (Mestrado) em Genética, Pontifícia Universidade Católica de Goiás, Goiânia, Goiás, Brazil; 4 Programa de Pós-Graduação em Biotecnologia e Biodiversidade, Universidade de Brasília, Brasília, DF, Brazil; 5 Laboratório de Citogenética Humana e Genética Molecular, Secretaria do Estado da Saúde de Goiás (LACEN/SESGO), Goiânia, GO, Brazil; 6 Programa de Pós-Graduação em Genética e Biologia Molecular, Laboratório de Genética e Biodiversidade, Universidade Federal de Goiás, Goiânia, GO, Brazil; CNRS UMR7275, France

## Abstract

Intellectual disability is a complex, variable, and heterogeneous disorder, representing a disabling condition diagnosed worldwide, and the etiologies are multiple and highly heterogeneous. Microscopic chromosomal abnormalities and well-characterized genetic conditions are the most common causes of intellectual disability. Chromosomal Microarray Analysis analyses have made it possible to identify putatively pathogenic copy number variation that could explain the molecular etiology of intellectual disability. The aim of the current study was to identify possible submicroscopic genomic alterations using a high-density chromosomal microarray in a retrospective cohort of patients with otherwise undiagnosable intellectual disabilities referred by doctors from the public health system in Central Brazil. The CytoScan HD technology was used to detect changes in the genome copy number variation of patients who had intellectual disability and a normal karyotype. The analysis detected 18 CNVs in 60% of patients. Pathogenic CNVs represented about 22%, so it was possible to propose the etiology of intellectual disability for these patients. Likely pathogenic and unknown clinical significance CNVs represented 28% and 50%, respectively. Inherited and *de novo* CNVs were equally distributed. We report the nature of CNVs in patients from Central Brazil, representing a population not yet screened by microarray technologies.

## Introduction

The most widely accepted definition of mental retardation (MD) relays on impairment of both cognitive and social adaptive functions [1]. Recently, the terminology MD had been replaced with DD/ID referring to patients affected with developmental delay/intellectual disability. Epidemiological data suggests that DD/ID affect up to 3% of the worldwide population and it may concur with or without multiple congenital anomalies (MCA) and also can be found in patients with no phenotypic traits [2]-[4].

ID is a complex, variable, and heterogeneous disorder, representing a disabling condition diagnosed worldwide, most commonly associated with neuropsychiatric disorder in children and adolescents [1]. ID is a condition characterized by the impairment of cognitive and adaptive skills that begins before the age of 18 years [3], [4]. The etiological diagnosis of ID remains a clinical challenge. For this particular trait, genetic and environmental factor may play an important role in its pathogenesis [1].

ID has a major impact on the life of the affected person, as well as his or her family and society. Thus, a specific diagnosis allows for a better understanding of the etiology of ID and patient clinical management, prognosis, and risk of recurrence [5]. Microscopic chromosomal abnormalities and well-characterized genetic conditions are the most common causes of ID, accounting for up to 40% of the observed cases. G-banding karyotyping is the standard genetic test for the laboratory investigation of ID and definitive diagnose is found in about 28% of ID cases following conventional karyotyping. Additionally, microscopic chromosome aberrations are found in association with up to 35% of the cases when using molecular cytogenetic tools, such as FISH, CGH, and MLPA that have contributed to increase the resolution of chromosomal rearrangement detection. However, about 50% of the molecular etiology of ID remains unknown. Therefore, at least half of children with ID remains without a diagnosis and poses a relevant clinical challenge [2], [6], [7].

Array-based chromosomal analyses have made it possible to identify putatively pathogenic copy number variation (CNVs) that could explain the molecular etiology of ID. Current estimates have indicated that approximately 15% to 20% of ID cases are due to submicroscopic CNVs [1], [8]–[10]. Recently, Chromosomal Microarray Analysis (CMA) using high density SNP probes increased genomic resolution and improved the detection of pathogenic microdeletions and microduplications. Moreover, the detection of cryptic genomic imbalances associated with apparently balanced chromosome rearrangements can also be detected when using CMA [11]. Nowadays, CMA has become the genetic test of choice to detect CNVs in patients with developmental disabilities and has increased the diagnostic yield for global developmental delay, intellectual disability, autism, and epilepsy [12], [13].

From 2010 through 2012, several patients with initial diagnosis of ID were referred to our laboratory. Herein, we attempt to establish an explanation for the phenotype of 15 patients with ID using a high-density resolution SNP microarray to determine clinical relevant CNVs over the entire genome. We report that 10/15 patients showed deletions and/or duplications that could explain patients' phenotype. To our knowledge, this is the first report on genomic rearrangements and ID in a cohort from Central Brazil, representing a population not yet screened by microarray technology.

The aim of the current study was to identify possible submicroscopic genomic alterations using a high-density chromosomal microarray in a retrospective cohort of patients with otherwise undiagnosable intellectual disabilities referred by doctors from the public health system in Central Brazil.

## Materials and Methods

### Patients

All participants had ID without etiological diagnosis after undergoing a thorough clinical evaluation. Assistant physicians from the Goiás state public health system referred each patient to our genetic service at both the Laboratory of Human Cytogenetic and Molecular Genetics and the Biology Department at Pontifical Catholic University in Central Brazil. The study population was comprised of a retrospective cohort which included 305 probands with clinical diagnosis of ID with our without multiple congenital anomalies (MCA) assisted at the Laboratory from 2010 to 2012. From those, 182 patients had visible chromosome aberrations from which accurate and definite diagnoses were possible. Of the remaining 123 cases, we contacted every family in order to explain about CMA as a new available genetic test potentially useful to explain the ID of the proband and also to offer the test to the family. From the remaining cases, 3 children died, 39 children missed one or both biological parents, for 44 cases the follow up were not successful due to address/telephone changes and additional information unavailability to contact the families, and 22 families were invited to participate in study. Based on an autonomous voluntary decision, only 15 families joined the task and signed an ethical informed consent if the cases adhered to the following inclusion criteria: (1) Normal G-banding karyotyping; (2) PCR negative results for *FMR1* gene mutations; (3) No family history of DD/ID; (4) No medical history of hypoxia, intoxication, infection or cranial trauma; (5) No history of perinatal brain injury; (6) Probands born to intellectually normal non-consanguineous parents; (7) Availability of biological parents to participate in the study. Due to cognitive limitations and/or individual age, probands were not able to make the decision to enroll themselves in the study. Thus, their parents or guardians signed the informed consent forms approved by the Ethics Committee on Human Research at the Pontifical Catholic University of Goiás (CEP-PUC/GO), under the protocol number 1721/2011.

### Biological Samples

For each proband and their biological parents, a total of 5 mL of peripheral blood was drawn using a standard vacuum extraction blood-collecting system containing EDTA. Genomic DNA was isolated from whole blood using QIAamp DNA Mini kit (Qiagen, Germany), following the manufacturer's instructions. Conventional cell cultures, harvesting, and G-banding at the level of 550 bands were performed for all patients following standardized procedures [14]. Chromosome observations were performed using a Zeiss Axioscope (Göttingen, Germany) and analyses using IKAROS (Metasystems Corporation, Altlussheim, Germany).

### CMA: Chromosomal Microarray Analysis

The analyses were carried out on probands and their biological parents in order to establish the origin of DNA rearrangements if *de novo* or inherited. A total of 250 ng of isolated DNA for each sample was digested with NspI, ligated, PCR amplified and purified, fragmented, biotin-labeled, and hybridized to be used in a GeneChip HD CytoScan Array (Affymetrix, Santa Clara, USA), following strictly the manufacturer's protocol to identify potentially pathogenic CNVs. The array was designed specifically for cytogenetic diagnose, including ∼2,7 million clinically relevant CNVs based on 743,304 SNP, and >1.9 million non-polymorphic probes covering the whole human genome. CEL files obtained by scanning the arrays were analyzed using the Chromosome Analysis Suite (ChAS) software (Affymetrix, Santa Clara, USA) in order to establish the genotypes. Genomic gains and losses that affected a minimum of 50 and 25 markers, respectively, in a 100 kb length were initially considered to determine the relevance of duplications and deletions. When using ChaS, CNVs boundaries were putatively inferred based on probe density. The two major quality control metrics for the GeneChip HD array were the Median Absolute Pairwise Difference (MAPD) and SNP-QC scores that apply to copy number and SNP probes, respectively. For our diagnostic setting, we applied the parameters ≤0.25 for MAPD and ≥15 for SNP-QC.

### CNV classification

CNVs were classified according to their nature, based on [2], [10], [15]. In summary, the CNVs found in each patient and their biological parents were compared with genomic variants in public databases, including Database of Genomic Variants (DGV), Database of Chromosomal Imbalance and Phenotype in Humans using Ensemble Resources (DECIPHER), and CytoScan HD Array Database. CNVs were classified as pathogenic, likely pathogenic, and of unknown clinical significance, according to [10], [15]. Benign CNVs were filtered out from subsequent analysis.

## Results

Herein, we report the results for CMA of 15 families, including proband and their biological parents. The cohort was comprised of 6 males and 9 females with intellectual disability with age ranging from 2 to 25 years old, and their progenitors with age at conception ranging from 21 to 47 years old. For all probands, G-banding karyotypes showed no visible alterations (46 XX or 46XY). The clinical and molecular features of patients were included in Table 1.

**Table 1:** Clinical and molecular features of 15 probands with intellectual disability screened with high-resolution CMA technology in Goás (Brazil).

Case	Clinical features*	Age (yo)	Sex	CNV	Mosaic (%)	Cytoband	Size (Mb)	Marker Count	Microarray nomenclature	Number of genes	Selected OMIN Morbid Genes**	Origin	Interpretation
001	GDD, SS	9	F	NAF									Negative***
002	GDD	11	M	Loss		1p13.3	0.13	54	1p13.3(108,726,456–108,853,796)x1	2	*NBPF4*	*de novo*	UCS
				Gain		1q44	0.53	600	1q44(246,174,090–246,702,392)x3	2	*SMYD3*	Inherited mat	UCS
				Gain		1q44	0.34	348	1q44(247,080,457–247,416,825)x3	8	*AHCTF1, ZNF124*	Inherited mat	UCS
				Gain	50	17p11.2	3.68	4151	17p11.2(16,769,800–20,446,820)x3	64	*COPS3, SMCR9, RAI1, SMCR5, TOM1L2, LRRC48, ATPAF2, DRG2, MYO15A, ALKBH5, FLII, SMCR8, SHMT1, USP32P2, CCDC144B, B9D1,MFAP4, RNF112*	*de novo*	Pathogenic
003	GDD	17	M	Gain		7q31.32	0.67	785	7q31.32(122,366,542–123,036,250)x3	3	*CADPS2*	Inherited pat	UCS
004	GDD, SS, MS	2	M	Gain		12q13.13.	0.52	396	12q13.13q13.2(54,462,464–54,980,062)x3	19	*SMUG1*	*de novo*	UCS
				Loss		14q11.2	0.21	298	14q11.2(22,732,618–22,941,375)x1	0		Inherited mat	UCS
005	GDD, MS	9	M	NAF									Negative
006	GDD	11	F	Loss		Xq27.3	4.18	10150	Xq27.3q28(144,580,614–148,757,072)x1	33	*CXorf1/TMEM257, FMR1, TMEM185A, IDS*	*de novo*	Pathogenic
007	GDD, MS, MCA	4	F	Loss		7q31.1	0.39	265	7q31.1(110,923,434–111,310,159)x1	1	*IMMP2L*	Inherited mat	LP
				Loss	30	18p11.32	1.23	1400	18p11.32(136,226–1,369,804)x1	11	*ADCYAP1*	*de novo*	LP
				Gain	40	18q11.1	5.80	2125	18q11.1q23(18,608,373–78,014,123)x2–3		*18q Partial Trissomy*	*de novo*	Pathogenic
				Gain		Xp22.33	25.72	31456	Xp22.33p21.3(168,546–25,887,307)x3	147	*NLGN4X, AP1S2, NHS, CDKL5, RPS6KA3, MBTPS2, SMS, ARX*	*de novo*	Pathogenic
008	GDD, SS	25	F	NAF									Negative
009	GDD, MS, ALS	10	F	NAF									Negative
010	GDD, MS	9	F	NAF									Negative
011	GDD, BD, MS	8	F	Loss		1p31.3	10.89	10080	1p31.3p31.1(68,693,129–79,580,916)x1	49	*AK5*	*de novo*	LP
012	GDD	6	M	Loss		14q11.2	0.34	580	14q11.2(22,599,355–22,943,573)x1	0		*de novo*	UCS
014	GDD, SS	14	M	Loss		14q11.2	0.14	188	14q11.2(22,799,790–22,944,507)x1	0		Inherited mat	UCS
				Gain		22q11.23	0.34	140	22q11.23q12.1(25,656,237–25,994,326)x3	4		Inherited mat	LP
015	GDD, MS	8	F	Loss		15q23	0.14	180	15q23(71,537,904–71,673,921)x1	1		Inherited mat	UCS
				Gain		Xq28	0.14	344	Xq28(152,720,466–152,860,955)x3	4	*ATP2B3, FAM58A*	Inherited mat	UCS
016	GDD, MS	5	F	NAF									Negative

* SS = Short Stature; GDD = Global Developmental Delay; MS = Multiple Stigmas, MCA = Multiple Congential Anomalies, ALS =  Autism Like Symptoms; BD = Behavior Disorders; Disturbance of brain electrical activity; ** Genes related to ID/Autsm; *** Negative means that no genomic rearrangements were found using CMA; yo = years old; NAF = No Alterations Found; UCS = Unknown Clinical Significance; LP = Likely Pathogenic.

**Figure 1:**
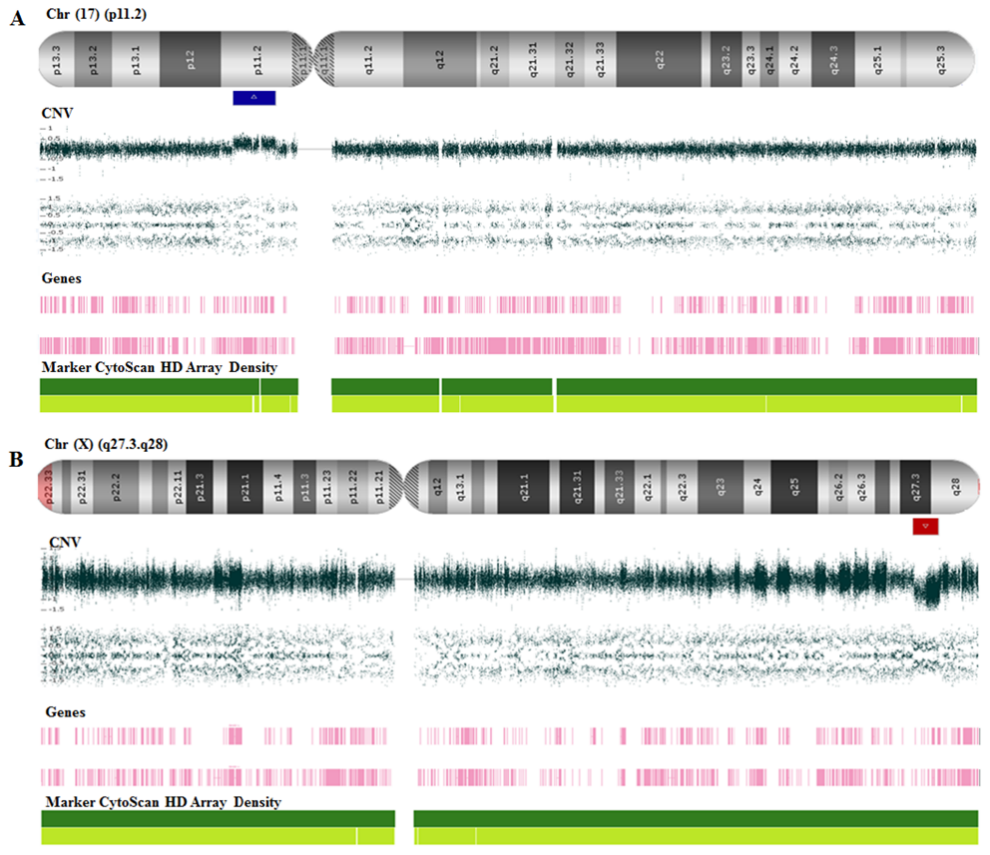
CNVs pathogenics from probands 002 and 006. (**A**) CMA from patient 002 showing a 3.677 Mb microduplication at 17p11.2 involving 64 genes. (**B**) CMA from patient 006 showing Xq27.3-q28 microduplication with 4.176 Mb that includes 4 genes related to intellectual disability.

**Figure 2:**
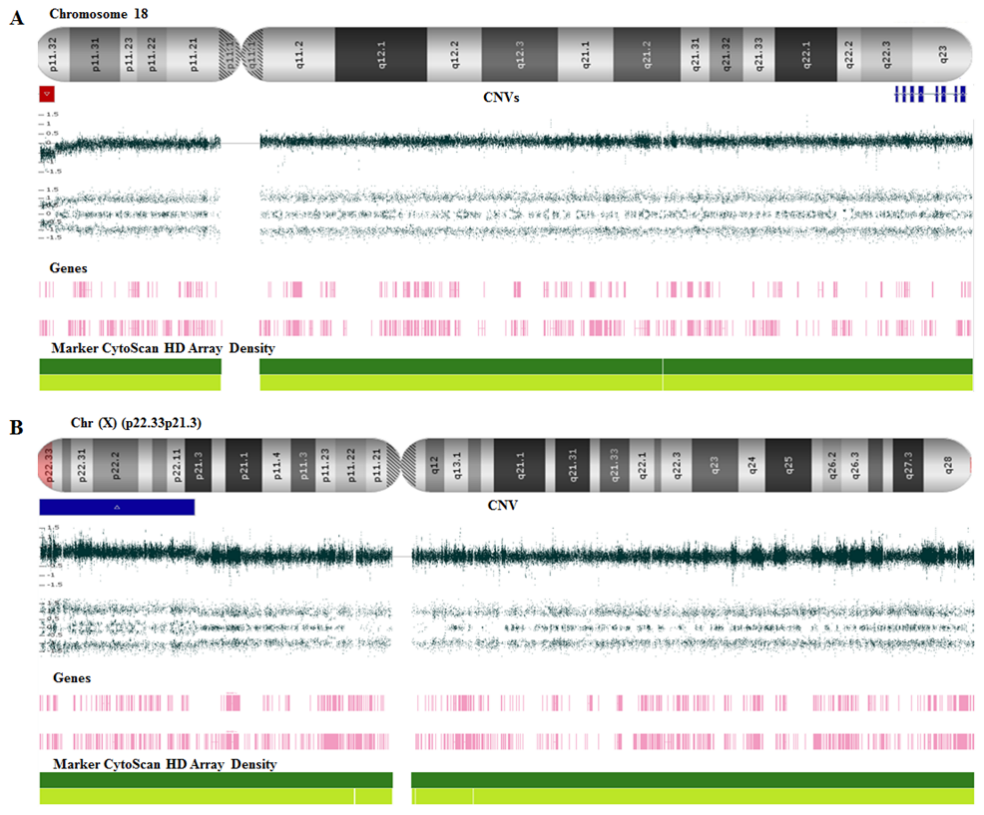
Genomic imbalances from proband 007. (**A**) 18p11.32 microdeletion (red line) and the 18q partial trisomy in mosaicismo (light blue line) (**B**) *de novo* microduplication at Xp22.33-Xp21.3 (blue line) that involves 147 genes.

We found a total of 18 CNVs that were identified in 9/15 (60%) patients. The CNV set was equally distributed, 9/18 (50%) and 9/18 (50%) were duplications and deletions, respectively. In six cases (40%), no chromosome rearrangement was observed. Moreover, molecular karyotyping of all 30 progenitors included in our cohort showed no evidence of chromosome rearrangements. Pathogenic and likely pathogenic CNVs were classified based on their size, gene content, and previously reported cases of potential involvement with pathogenic mechanism in human and animal models. In our cohort, pathogenic CNVs represented about 22% (4/18) of all observed rearrangements and were associated with chromosomes 17, 18, and X (Figure 1 and Figure 2), involving genes that were related to the formation and/or maintenance of the central nervous system. Also, pathogenic CNVs included morbid genes from OMIM (Online Mendelian Inheritance in Man). Moreover, all pathogenic CNVs in this study had a *de novo* origin. Four out of 18 (22%) CNVs were classified as likely pathogenic and 10/18 (56%) CNVs were classified as of unknown clinical significance because they overlapped by more than 90% of the CNVs observed in the databases of normal control groups. Inherited and *de novo* CNVs were equally distributed. However, 8/18 (45%) of CNVs were maternally inherited.

## Discussion

The correct diagnosis of a neurological disorder is crucial for predicting the probands' clinical follow up, to establish accurate prognostic, and to provide adequate genetic counseling. CMA technology is a relatively new strategy useful as an additional tool for genetic diagnosis. The method has been recommended as the first-tier diagnostic test for patients with global developmental delay, intellectual disability, autism spectrum disorders, and multiple congenital anomalies [13].

In the current study, high-resolution CMA was carried out on 15 patients with ID, and relevant CNVs were found in 9 patients. From those, it was possible to propose the etiology of intellectual disability for 3 patients (20%), which is consistent with other studies that have used this or similar technologies, and reported improving the diagnostic yield up to 10–25% [10], [15]–[17].

No changes in copy number were observed in six (40%) patients. Thus, it was not possible to suggest a genetic cause for the ID and high-resolution CMA was not useful to diagnose these cases. The possibility of testing these individuals with more sensitive or specific genomic technologies, such as next-generation exome sequencing could be a fruitful diagnostic approach in order to identify gene mutations that may be causing the observed phenotype [18]–[20]. Moreover, ID is a polygenic complex and heterogeneous multifactorial trait, which remains a diagnostic challenge for human geneticists and heavily affected by environmental factors.

We identified 4 (22%) pathogenic CNVs, including chromosomal imbalances associated with 17p11.2, Xq27.3, 18q11.1 and Xp22.33. Case 2 was a boy who presented a *de novo* microduplication at 17p11.2. Interestingly, the log ratio probe intensity on this region was compatible with a mosaic duplication affecting about 50% of cells. The region has been implicated in the Potocki-Lupski Syndrome (MIM 610883) [21], [22]. Additionally, microdeletion of this region has been associated with Smith-Magenis Syndrome (MIM 182290). Gain or loss of genomic material on chromosome band 17p11.2 inevitably leads to phenotypes that include ID as a relevant trait [23].

Patient 6 was a girl who showed a *de novo* microdeletion of about 4.2 Mb at Xq27.3, including the region of the Fragile X Syndrome (FXS). CGG trinucleotide repeats expansions are the most common cause of FXS. However, less frequently point mutations and partial or full deletions of the *FMR1* gene also cause the FXS. To date, only 10 female index patients with deletions harboring *FMR1* have been reported. Moreover, the severity of the phenotype for females with *FMR1* deletions correlates with their X-chromosome inactivation [23]–[25].

Case 7 was a girl severely affected with multiple congenital abnormalities and intense dysmorphology. This proband, presented a *de novo* 18q partial trisomy with 40% mosaicism identified by CMA. Furthermore, we analyzed 100 metaphase spreads and we observed the duplication of 18q in a mosaic state in 45% of the cells. We also found that the break point called by the CMAs was overestimated due to an array of small duplications upstream from the real duplication site, which created an artificially large partial trisomy of 18. Also we observed in CMA a small deletion in the terminal region stating at 18p11.32. The karyotyping reveled that in fact there was a duplication of a distal region of 18q starting at 18q22.3, resulting in a segment of 5.8 Mb. The chromosome images revealed that the two breakpoints alongside the chromosome 18 created a rearranged chromosome, which included a pericentric inversion of that DNA segment. As a result, there was a loss of the terminal end of 18q, including its centromere. Although CMA was robust to identify the deletion and the duplication, it was not able to indicate the chromosomal inversion. Moreover, especial care must be devoted to the callings made CMA in order to avoid overestimation of the size of the segments involved in gain or loss of chromosome material.

Full trisomy 18 is known to cause Edward's Syndrome. However, partial trisomy of chromosome 18, especially involving 18q, has long been reported to cause peculiar phenotypes that show cognitive impairment, varying from a milder to a more severe form, with or without internal malformation. The extent of the partial trisomy and the level of the mosaic state affect the odds of patients' survival [26]–[31]. Interstitial duplications of the short arm of the X chromosome have been rarely described [32]. In Patient 7, we also observed a *de novo* microduplication at Xp22.33-Xp21.3 harboring 8 OMIM morbid genes (Table 1) that have been described in association with ID [32]–[36].

The CNVs designated as likely pathogenic comprise of 22% of our data. They were called likely pathogenic because they harbor genes having well-established association with abnormal phenotypes. Moreover, their genic content has been implicated in the process of neurological development (42), as mediators of neuroendocrine stress responses (51), to be expressed exclusively in the brain (64). However, none of the genes observed in the likely pathogenic CNVs was yet directly related to ID. We also observed a maternally inherited region which was involved with Chromosome 22q11.2 Duplication Syndrome (MIM 608363) [37]. This region is also found deleted in the DiGeorge Syndrome (MIM 188400) and Emanuel Syndrome (MIM 609029). Both syndromes are characterized by multiple congenital anomalies, significant developmental delay, and mental retardation [38].

At the case 15, despite the location of *ATP2B3* and *FAM58A* genes in Xq28, this region has not yet been implicated in ID *per se*. *ATP2B3* gene encodes a calcium-transporting ATPase predominantly expressed in the brain, and mutations in the gene have been associated with increased plasmatic concentrations of aldosterone and reduced plasmatic potassium [39]. Moreover, base substitution in *ATP2B3* identified by exome sequencing in a family with X-linked congenital ataxia (XCA) indicated the importance of calcium homeostasis in neurons. Nevertheless, the affected persons present neither mental retardation nor pyramidal tract involvement at their neurological examinations [40]. On the other hand, mutations in *FAM58A* cause an X-linked dominant disorder known as STAR Syndrome (MIM 300707). This syndrome presents facial dimorphism, toe syndactyly, telecanthus, anogenital and renal malformations [41]. Nevertheless, patients with STAR Syndrome do not show ID.

The proportion of CNVs classified as of unknown clinical significance was high (56%) in our study. According to researches, the ability to detect CNVs has far outpaced our ability to understand their role in a disease [16]. Inheritance studies are the primary strategy recommended to estimate the role of such CNVs in pathogenicity. Nevertheless, it is often imprudent to attribute clinical significance to a CNV based solely on its inheritance pattern as a growing number of CNVs show an incomplete penetrance and also because de novo CNVs may represent benign variants. The clinical and genetic interpretation of the data acquired by CMA technologies still remains a challenge and often require further specific investigations [42]. To confirm the pathogenicity of a CNV requires studies designed to understand the causative relation between the genomic imbalances to the investigated disease. The genetic interpretation and clinical relevance of the data acquired by CMA technologies remain a challenge. Primarily due to the deleterious effects caused by small genomic variations, such as point mutations, further influenced by reduced penetrance, variable expressivity, and gene dosage [43].

Despite the small size of the cohort screened in the current study, here we report the nature of CNVs in patients from Central Brazil, representing a population not yet screened by microarray technologies. Tests based on microarray technologies are relatively new and are likely to continue to evolve in the coming years. For both, patients and families, it is very important to establish a diagnosis for ID. Furthermore, the diagnosis has to be linked to genetic counseling to ensure that parent's views and preferences are taken into account, following a non-directive approach. The results of our study helped the families and their assistant physicians to reach an accurate diagnosis bringing closure to their post-natal search for an explanation regarding the ID observed in the family.

## References

[1] Wu CL, Lin JD, Hu J, Yen CF, Yen CT, et al (2010) The effectiveness of healthy physical fitness programs on people with intellectual disabilities living in a disability institution: six-month short-term effect. Res Dev Disabil 31(3): 713–7 10.1016/j.ridd.2010.01.013 20172687

[2] Pani AM, Hobart HH, Morris CA, Mervis CB, Bray-Ward P, et al (2010) Genome rearrangements detected by SNP microarrays in individuals with intellectual disability referred with possible Williams syndrome. PLoS One 31 5(8): e12349 10.1371/journal.pone.0012349 PMC2930846 20824207

[3] Millichap JG (2003) Evaluation of Global Developmental Delay. AAP Grand Rounds 9: 62–63 10.1542/gr.9-6-62

[4] Galasso C, Lo-Castro A, El-Malhany N, Curatolo P (2010) “Idiopathic” mental retardation and new chromosomal abnormalities. Ital J Pediatr 36: 17 10.1186/1824-7288-36-17 20152051 PMC2844383

[5] Pfundt R, Veltman JA (2012) Structural genomic variation in intellectual disability. Methods Mol Biol 838: 77–95 10.1007/978-1-61779-507-73 22228007

[6] Flore LA, Milunsky JM (2012) Updates in the genetic evaluation of the child with global developmental delay or intellectual disability. Semin Pediatr Neurol 19: 173–180 10.1016/j.spen.2012.09.004 23245550

[7] Rauch A, Hoyer J, Guth S, Zweier C, Kraus C, et al (2006) Diagnostic yield of various genetic approaches in patients with unexplained developmental delay or mental retardation. Am J Med Genet A 140: 2063–2074 10.1002/ajmg.a.31416 16917849

[8] Gijsbers AC, Lew JY, Bosch CA, Schuurs-Hoeijmakers JH, van Haeringen A, et al (2009) A new diagnostic workflow for patients with mental retardation and/or multiple congenital abnormalities: test arrays first. Eur J Hum Genet 17: 1394–1402 10.1038/ejhg.2009.74 19436329 PMC2986688

[9] Zahir F, Friedman JM (2007) The impact of array genomic hybridization on mental retardation research: a review of current technologies and their clinical utility. Clin Genet 72: 271–287 10.1111/j.1399-0004.2007.00847.x 17850622

[10] Miller DT, Adam MP, Aradhya S, Biesecker LG, Brothman AR, et al.. (2010) Consensus Statement: Chromosomal Microarray Is a First-Tier Clinical Diagnostic Test for Individuals with Developmental Disabilities or Congenital Anomalies. Am J Med Genet 86, 749–764. doi: 10.1016/j.ajhg.2010.04.006 10.1016/j.ajhg.2010.04.006 PMC2869000 20466091

[11] Cooper GM, Coe BP, Girirajan S, Rosenfeld JA, Vu TH, et al (2011) A copy number variation morbidity map of developmental delay. Nat Genet 43: 838–846 10.1038/ng.909 21841781 PMC3171215

[12] McGillivray G, Rosenfeld JA, McKinlay Gardner RJ, Gillam LH (2012) Genetic counselling and ethical issues with chromosome microarray analysis in prenatal testing. Prenat Diagn 32: 389–395 10.1002/pd.3849 22467169

[13] Riggs E, Wain K, Riethmaier D, Smith-Packard B, Faucett W, et al.. (2013) Chromosomal microarray impacts clinical management. Clin Genet. Jan 25. doi: 10.1111 10.1111/cge.12107 23347240

[14] Verma RS, Babu A (1995) Human chromosomes Principles and Techiniques. Second edition. (MacGraw-Hill).

[15] Battaglia A, Doccini V, Bernardini L, Novelli A, Loddo S, et al (2013) Confirmation of chromosomal microarray as a first-tier clinical diagnostic test for individuals with developmental delay, intellectual disability, autism spectrum disorders and dysmorphic features. Eur J Paediatr Neurol 17(6): 589–99 doi:10.1016 23711909 10.1016/j.ejpn.2013.04.010

[16] Zilina O, Teek R, Tammur P, Kuuse K, Yakoreva M, et al (2014) Chromosomal microarray analysis as a first-tier clinical diagnostic test: Estonian experience. Mol Genet Genomic Med 2(2): 166–75 10.1002/mgg3.57 24689080 PMC3960059

[17] Howell KB, Kornberg AJ, Harvey AS, Ryan MM, Mackay MT, et al (2013) High resolution chromosomal microarray in undiagnosed neurological disorders. J Paediatr Child Health 49(9): 716–24 10.1111/jpc.12256 23731025

[18] Lalonde E, Albrecht S, Ha KC, Jacob K, Bolduc N, et al (2010) Unexpected allelic heterogeneity and spectrum of mutations in Fowler syndrome revealed by next-generation exome sequencing. Hum Mutat 31: 918–923 10.1002/humu.21293 20518025

[19] Ku CS, Naidoo N, Pawitan Y (2011) Revisiting Mendelian disorders through exome sequencing. Hum Genet 129: 351–370 10.1007/s00439-011-0964-2 21331778

[20] Toscano CD, Guilarte TR (2005) Lead neurotoxicity: from exposure to molecular effects. Brain Res Brain Res Rev 49: 529–554 10.1016/j.brainresrev.2005.02.004 16269318

[21] Potocki L, Bi W, Treadwell-Deering D, Carvalho CM, Eifert A, et al (2007) Characterization of Potocki-Lupski syndrome (dup(17)(p11.2p11.2)) and delineation of a dosage-sensitive critical interval that can convey an autism phenotype. Am J Hum Genet 80: 633–649 10.1086/512864 17357070 PMC1852712

[22] Treadwell-Deering DE, Powell MP, Potocki L (2010) Cognitive and behavioral characterization of the Potocki-Lupski syndrome (duplication 17p11.2). J Dev Behav Pediatr 31: 137–143 10.1097/DBP.0b013e3181cda67e 20110824

[23] Dahl N, Hu LJ, Chery M, Fardeau M, Gilgenkrantz S, et al (1995) Myotubular myopathy in a girl with a deletion at Xq27-q28 and unbalanced X inactivation assigns the MTM1 gene to a 600-kb region. Am J Hum Genet 56(5): 1108–15. 7726166 PMC1801465

[24] Probst FJ, Roeder ER, Enciso VB, Ou Z, Cooper ML, et al (2007) Chromosomal microarray analysis (CMA) detects a large X chromosome deletion including FMR1, FMR2, and IDS in a female patient with mental retardation. Am J Med Genet A. 15 143A(12): 1358–65. 10.1002/ajmg.a.31781 17506108

[25] Zink AM, Wohlleber E, Engels H, Rødningen OK, Ravn K, et al (2014) Microdeletions including FMR1 in three female patients with intellectual disability - further delineation of the phenotype and expression studies. Mol Syndromo 5(2): 65–75 10.1159/000357962 PMC3977317 24715853

[26] Freson K, Hashimoto H, Thys C, Wittevrongel C, Danloy S, et al (2004) The pituitary adenylate cyclase-activating polypeptide is a physiological inhibitor of platelet activation. J Clin Invest 113: 905–912 10.1172/JCI19252 15067323 PMC362113

[27] Fryns JP, Detavernier F, van Fleteren A, van den Berghe H (1978) Partial trisomy 18q in a newborn with typical 18 trisomy phenotype. Hum Genet 44: 201–205 10.1007/BF00295415 730164

[28] Turleau C, Chavin-Colin F, Narbouton R, Asensi D, Grouchy JD (1980) Trisomy 18q-. Trisomy mapping of chromosome 18 revisited. Clinical Genet 18: 20–26 10.1111/j.1399-0004.1980.tb01359.x 7418250

[29] de Muelenaere A, Fryns JP, van den Berghe H (1981) Familial partial distal 18q (18q22-18q23) trisomy. Ann Genet 24: 184–186. 6974534

[30] Mewar R, Kline AD, Harrison W, Rojas K, Greenberg F, et al (1993) Clinical and Molecular Evaluation of Four Patients with Partial Duplications of the Long Arm of Chromosome 18. Am J Hum Genet 53: 1269–1278. 8250043 PMC1682493

[31] Boghosian-Sell L, Mewar R, Harrison W, Shapiro RM, Zackai EH, et al (1994) Molecular mapping of the Edwards syndrome phenotype to two noncontiguous regions on chromosome 18. Am J Hum Genet 55: 476–483. 8079991 PMC1918415

[32] Daoud H, Bonnet-Brilhault F, Védrine S, Demattéi MV, Vourc'h P, et al (2009) Autism and nonsyndromic mental retardation associated with a de novo mutation in the NLGN4X gene promoter causing an increased expression level. Biol Psychiatry 66(10): 906–10 10.1016/j.biopsych.2009.05.008 19545860

[33] Tarpey PS, Stevens C, Teague J, Edkins S, O'Meara S, et al (2006) Mutations in the gene encoding the Sigma 2 subunit of the adaptor protein 1 complex, AP1S2, cause X-linked mental retardation. Am J Hum Genet 79(6): 1119–24. 17186471 10.1086/510137 PMC1698718

[34] Chograni M, Rejeb I, Jemaa LB, Châabouni M, Bouhamed HC (2011) The first missense mutation of NHS gene in a Tunisian family with clinical features of NHS syndrome including cardiac anomaly. Eur J Hum Genet 19(8): 851–6 10.1038/ejhg.2011.52 21559051 PMC3172933

[35] Hagebeuk EE, van den Bossche RA, de Weerd AW (2013) Respiratory and sleep disorders in female children with atypical Rett syndrome caused by mutations in the CDKL5 gene. Dev Med Child Neurol 55(5): 480–4 10.1111/j.1469-8749.2012.04432.x 23151060

[36] Das DK, Mehta B, Menon SR, Raha S, Udani V (2013) Novel mutations in cyclin-dependent kinase-like 5 (CDKL5) gene in Indian cases of Rett syndrome. Neuromolecular Med 15(1): 218–25 10.1007/s12017-012-8212-z 23242510

[37] Brunet A, Gabau E, Perich RM, Valdesoiro L, Brun C, et al (2006) Microdeletion and microduplication 22q11.2 screening in 295 patients with clinical features of DiGeorge/Velocardiofacial syndrome. Am J Med Genet A 140(22): 2426–32. 17041934 10.1002/ajmg.a.31499

[38] Carter MT, St Pierre SA, Zackai EH, Emanuel BS, Boycott KM (2009) Phenotypic delineation of Emanuel syndrome (supernumerary derivative 22 syndrome): Clinical features of 63 individuals. Am J Med Genet A 149A(8): 1712–21 doi:10.1002 19606488 10.1002/ajmg.a.32957 PMC2733334

[39] Beuschlein F, Boulkroun S, Osswald A, Wieland T, Nielsen HN, et al.. (2013) Somatic mutations in ATP1A1 and ATP2B3 lead to aldosterone-producing adenomas and secondary hypertension. Nat Genet. 45(4):440–4, 444e1-2. doi: 10.1038/ng.2550 10.1038/ng.2550 23416519

[40] Bertini E1, des Portes V, Zanni G, Santorelli F, Dionisi-Vici C, et al (2000) X-linked congenital ataxia: a clinical and genetic study. Am J Med Genet 1 92(1): 53–6. 10797423

[41] Unger S1, Böhm D, Kaiser FJ, Kaulfuss S, Borozdin W, et al (2008) Mutations in the cyclin family member FAM58A cause an X-linked dominant disorder characterized by syndactyly, telecanthus and anogenital and renal malformations. Nat Genet 40(3): 287–9 10.1038/ng.86 18297069

[42] Kearney HM, South ST, Wolff DJ, Lamb A, Hamosh A, et al (2011) American College of Medical Genetics recommendations for the design and performance expectations for clinical genomic copy number microarrays intended for use in the postnatal setting for detection of constitutional abnormalities. Genet Med 13(7): 676–9 10.1097/GIM.0b013e31822272ac 21681105

[43] Zanni G, Calì T, Kalscheuer VM, Ottolini D, Barresi S, et al (2012) Mutation of plasma membrane Ca2+ ATPase isoform 3 in a family with X-linked congenital cerebellar ataxia impairs Ca2+ homeostasis. Proc Natl Acad Sci U S A 109: 14514–14519 10.1073/pnas.1207488109 22912398 PMC3437887

